# Thyroid function modifies the association between ratio of triglyceride to high-density lipoprotein cholesterol and renal function: a multicenter cross-sectional study

**DOI:** 10.1038/srep11052

**Published:** 2015-07-16

**Authors:** Zhongshang Yuan, Meng Zhao, Bingchang Zhang, Haiqing Zhang, Xu Zhang, Qingbo Guan, Guang Ning, Ling Gao, Fuzhong Xue, Jiajun Zhao

**Affiliations:** 1 Department of Epidemiology and Biostatistics, School of Public Health, Shandong University, Jinan, Shandong, China; 2 Department of Endocrinology and Metabolism, Shandong Provincial Hospital affiliated to Shandong University, Jinan, Shandong, China; 3 Institute of Endocrinology and Metabolic Diseases, Shandong Academy of Clinical Medicine, Jinan, Shandong, China; 4 Clinical Laboratory, Shandong Provincial Hospital affiliated to Shandong University, Jinan, Shandong, China; 5 Shanghai Clinical Center for Endocrine and Metabolic Diseases, Shanghai Institute of Endocrine and Metabolic Diseases, Department of Endocrine and Metabolic Diseases, Rui-Jin Hospital, Shanghai Jiao Tong University School of Medicine, Shanghai, China; 6 Scientific Center, Shandong Provincial Hospital affiliated to Shandong University, Jinan, Shandong, China

## Abstract

Hypothyroidism was confirmed to be associated with both dyslipidemia and renal dysfunction. However, the impact of thyroid function on the relationship between serum lipid levels and renal function has never been given sufficient attention. In this large-scale multicenter cross-sectional study, the ratio of triglyceride to high-density lipoprotein cholesterol (TG/HDL) and the prevalence of hypothyroidism in CKD subjects were significantly higher than those in non-CKD ones (*P* < 0.001). After adjustment for potential confounding factors, TG/HDL was shown to be significantly associated with serum Cr levels (β = 0.551; 95%CI, 0.394–0.708), and eGFR (β = −0.481; 95%CI, −0.731–−0.230). The risk for CKD was significantly increased as TG/HDL ratio was elevated (adjusted odds ratio = 1.20; 95%CI, 1.11–1.27). These significant associations were found among subjects with euthyroidism and hypothyroidism rather than hyperthyroidism. Furthermore, the associations between TG/HDL and Cr or CKD status were significantly greater in hypothyroidism than those in euthyroidism (*P* < 0.05). These results suggested that elevated TG/HDL ratio was associated with renal dysfunction; it exhibited a significantly stronger association with Cr and CKD in hypothyroidism than in euthyroidism. Therefore, more attention should be paid on lipid profile to prevent or delay the occurrence and progression of renal dysfunction, especially for those with hypothyroidism.

Renal dysfunction is becoming a serious public health issue worldwide due to its high prevalence and significant association with the morbidity and mortality of many fatal chronic diseases^1^,^2^. It is highly desirable to identify and manage the risk factors associated with renal dysfunction. Evidence suggesting that abnormal lipid metabolism may be a possible cause of kidney dysfunction has mounted significantly in recent years^3^,^4^. High triglyceride (TG) levels and/or low high-density lipoprotein cholesterol (HDL-C) levels were illustrated to predict an increased risk of renal dysfunction in many studies^5^,^6^,^7^. Recently, the ratio of TG/HDL, a more reasonable indicator of insulin resistance and cardiovascular events, has increasingly drawn researchers’ attention^8^. Actually, the association between TG/HDL and chronic kidney disease (CKD) has been confirmed in Korean adults^9^ and Japanese population^4^, and it was also shown to be the unique lipid-related ratio independently associated with CKD stage 3 or more^10^. Nevertheless, none of the above researches considered the other confounding lipid metabolism factors including total cholesterol and low-density lipoprotein cholesterol in their statistical analyses. Furthermore, few attempts were systematically conducted, especially for large-scale Chinese population, to explore the association between TG/HDL and the renal function parameters including serum creatinine (Cr), estimated glomerular filtration rate (eGFR) and CKD status.

On the other hand, hypothyroidism, the most common thyroid dysfunction^11^, has been confirmed to be associated with both dyslipidemia and renal dysfunction in a lot of epidemiological studies^7^,^11^,^12^,^13^. However, it remains poorly elucidated whether thyroid function has impact on the relationship between TG/HDL and renal function. Sometimes even when exposed to the same TG/HDL level, renal function might be different from one individual to another according to different thyroid function status. Some subjects could experience marked changes in renal function, while others were unresponsive. Thus, the interactions between TG/HDL and thyroid function might play important roles in the pathogenesis of renal dysfunction and should be further explored.

Thus, based on REACTION study, we first systematically detected the association between TG/HDL and the renal function parameters including Cr, eGFR and CKD status, and further ascertained the impact of thyroid function on this relationship in a large multicenter Chinese population. Our results may have important clinical implications for guidelines aiming at preventing renal disease.

## Results

### Clinical characteristics of the study population

Table 1 showed the clinical characteristics of the 22,133 participants (7,806 men and 14,327 women), with 268 subjects having CKD (the prevalence was 1.2%). Among participants with CKD, the median of TG/HDL was significantly higher and the prevalence of hypothyroidism was elevated. In addition, the CKD subjects had older age, higher percentage of female participants, increased blood pressure, elevated serum levels of lipid profiles, fasting plasma glucose and aspartate aminotrasferase.

### The association between TG/HDL and renal function

Table 2 presented the univariate and multivariate association between TG/HDL and Cr, eGFR and risk of CKD in the linear mixed model (for Cr and eGFR) and generalized linear mixed model (for CKD status). As shown in Table 2, after adjustment for potential confounding factors, TG/HDL ratio was positively correlated with serum Cr levels (β = 0.551; 95%CI, 0.394–0.708), and there was significantly negative association between TG/HDL ratio and eGFR (β = −0.481; 95%CI, −0.731–−0.230). Consistently, the risk for CKD was increased as TG/HDL ratio was elevated (odds ratio = exp (β) = 1.20; 95%CI, 1.11–1.27). Subgroup analysis showed that the associations between TG/HDL and eGFR and CKD disappeared in males, while all adjusted associations between TG/HDL and the three renal function parameters could also be significantly found in the other three subgroups (Table S1-S4).

### The association between TG/HDL and renal function according to thyroid function status

**Figure 1:**
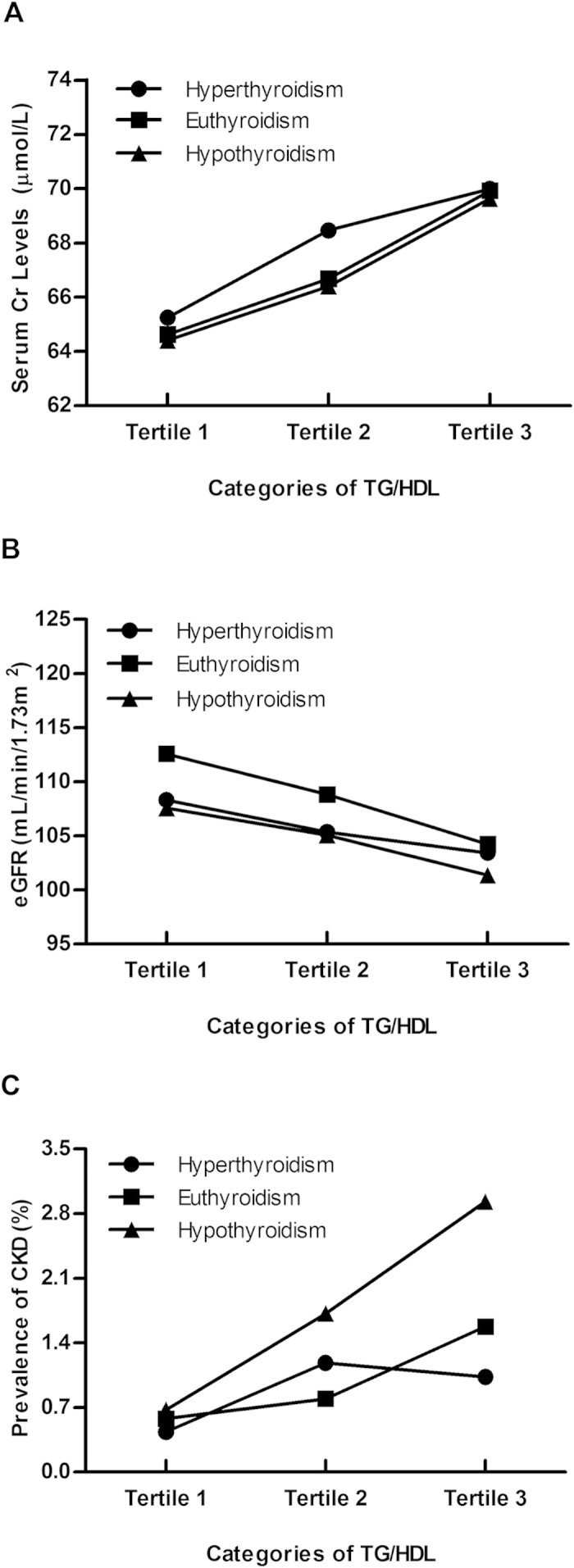
Correlation of TG/HDL with renal function according to thyroid function status. The data are presented as the means (Cr and eGFR) or prevalence (CKD status). The tertile ranges are as follows: Tertile 1 (<0.79), Tertile 2 (0.79–1.38), Tertile 3 (>1.38). TG, triglyceride; HDL, high-density lipoprotein; Cr, creatinine; eGFR, estimated glomerular filtration rate; CKD, chronic kidney diseases.

To determine the presence of Cr, eGFR and CKD status across the spectrum of TG/HDL, we stratified TG/HDL into three groups (tertiles) in different thyroid function status. As shown in Fig. 1 and Table 3, following the elevated TG/HDL, the trends of eGFR were gradually decreased in both euthyroidism (β = −1.37, *P* < 0.001) and hypothyroidism (β = −1.48, *P* < 0.001), respectively, but the trend in hyperthyroidism (β = −0.55, *P* = 0.31) was different from those in euthyroidism and hypothyroidism. Analogously, the trends for Cr and CKD status were also similar in euthyroidism and hypothyroidism but different from that in hyperthyroidism. Further adjusted investigation of the association between TG/HDL and renal function according to thyroid function status was shown in Table 3. TG/HDL ratio was not significantly correlated with all three renal function parameters among subjects with hyperthyroidism. The same phenomenon was observed in subgroup analysis (Table S5-S8). Significant associations emerged in euthyroidism and hypothyroidism, though the *P* value for eGFR in hypothyroidism was a little higher (*P* = 0.06) than given significance level. However, subgroup analysis illustrated that this phenomenon only existed in females and also in the group with age older than 65 years (Table S5-S8). Furthermore, compared to euthyroidism, the associations between TG/HDL and Cr and CKD status were significantly greater in subjects with hypothyroidism.

## Discussion

In this large-scale multicenter cross-sectional study, we first demonstrated that TG/HDL ratio was independently associated with Cr, eGFR and risk of CKD after adjustment of potential confounding factors. Then we confirmed that the associations between TG/HDL and renal function were greater in subjects with hypothyroidism than euthyroidism, while the significant association was not observed in hyperthyroidism. Our study indicated that TG/HDL ratio was associated with renal function, and this association could be modified by thyroid function status.

As a considerably important lipid ratio, TG/HDL was used as a surrogate for small density LDL-C and insulin resistance, and predicted coronary heart disease independently^14^,^15^. For the association between TG/HDL and renal function, several studies had obtained results similar to ours. Tsuruya K *et al*. observed that the prevalence of CKD, low eGFR, and proteinuria increased significantly with elevating quartiles of TG/HDL-C in both genders in a large Japanese population^4^. Similarly, Kim J.Y. *et al*. showed that the prevalence with CKD stage 3 or more increased with increasing quartile group in both sexes in Koreans^8^. Remarkably, a follow-up study demonstrated that the TG/HDL-C ratio was positively associated with an increased risk of incident CKD in type 2 diabetes mellitus patients^16^. However, the above studies didn’t consider the other confounding lipid metabolism factors including total cholesterol and low-density lipoprotein cholesterol in their statistical analyses, or were performed in a specific population (for example, type 2 diabetes mellitus patients). In this study, we performed the analyses in a general population and observed the association between TG/HDL ratio and renal function after adjustment for potential confounding factors including serum cholesterol levels and thyroid function.

Another important finding for our present study was that the association between TG/HDL and renal function would become greater in hypothyroidism. Although intensive mechanism was not fully elucidated, hypothyroidism was confirmed to be closely associated with both dyslipidemia and renal dysfunction. In the Colorado research, lipid levels increased in a graded fashion as thyroid function declined^12^. In the HUNT study, Asvold B.O. *et al*. demonstrated that CKD was more common in subjects with hypothyroidism; even within the reference range, the prevalence of CKD was gradually increased as serum thyrotropin levels increased^13^. It seems quite clear that both renal function and lipid metabolism could be modulated by thyroid function. However, so far, no previous works paid attention to the influence of thyroid function on the relationship between TG/HDL and renal function. The present study illustrated the modification of thyroid function on the association between TG/HDL and renal function, suggesting that more attention should be paid to lipid profiles to prevent or delay the occurrence and progression of renal dysfunction, especially for those with hypothyroidism. Further laboratory studies are also essential to determine the involved mechanism.

In this large-scale multicenter study, a mixed model was employed to investigate the association between lipid and renal function. The mixed model is particularly useful in settings where measurements are made on clusters of related statistical units and subjects are observed nested within larger units. Within-cluster correlation is more likely to be captured in mixed model through adding random cluster effects into the traditional regression model^17^,^18^. In our study, data were collected from three different provinces (clusters) and subjects within each cluster were obviously correlated, thus mixed model was preferred. Further analysis showed that the estimated standard deviation of the random effects for Cr, eGFR and risk of CKD was 3.29, 7.01 and 1.39 respectively (data not shown), and all values were significantly higher than zero (*P* < 0.001), indicating that it was indeed necessary to incorporate random effects in the model.

Previous studies have provided strong evidence suggesting that lipid abnormality might lead to kidney dysfunction. *In vivo* studies, Levi M. *et al*. demonstrated that in mice fed a high-fat diet, lipids accumulated in the glomerular and tubulo-interstitial cells with increased expression of sterol regulatory element binding protein (SREBP), a transcription factor which plays a key role in lipogenesis. These changes were associated with significant glomerulosclerosis and proteinuria^19^,^20^. In addition, Tanaka Y. *et al*. showed a protecting effect of fenofibrate (a potent PPAR-α agonist) reducing glomerular lipid accumulation and oxidative stress associated with decreased albuminuria and glomerulosclerosis in mice fed a high-fat diet^21^. Further more, *in vitro* studies indicated that the mechanisms associated with lipid induced kidney injury may be linked to insulin resistance. The blocking of insulin signaling in podocytes and proximal tubular cells induced by palmitic acid or insulin receptor deletion may finally contribute to the renal dysfunction^22^,^23^. On the other hand, while several causes were determined to result in decreased GFR, the involved mechanisms have not been fully elucidated. Firstly, hypothyroidism is associated with pathophysiological status which could lead to decreased renal perfusion, including decreased cardiac output and circulating volume, impaired activity of the renin-angiotensin-aldosterone system (RAAS), and a decreased atrial natriuretic peptide (ANP) level^24^,^25^,^26^. Secondly, growth retardation in the parenchyma of the kidney may lead to the decreased glomerular surface area^27^. Thirdly, in the proximal tubule, a filtrate overload caused by deficient sodium and water reabsorption could result in adaptive preglomerular vasoconstriction^28^. Fourthly, in the distal tubule, the decreased expression of the chloride channel ClC-2 contributed to the declined GFR by the tubulo-glomerular feedback^29^. Finally, a reduction of the expression of several glomerular vasodilators, including insulin-like growth factor 1 and vascular endothelial growth factor, might cause the reduction of GFR^30^. From the above, both lipid abnormality and hypothyroidism could result in renal dysfunction; this might be a plausible explanation for the greater effect of TG/HDL on renal function in subjects with hypothyroidism in the present study.

The prevalence of CKD based on eGFR was about 1.2% in the present study, which was lower than that in US population^31^. However, this prevalence was similar to that reported in the first national survey of CKD across China (1.7%)^32^. One plausible explanation for the lower prevalence of decreased eGFR in China might be that hypertension and diabetes have increased rapidly in the past 15–20 years in China, but for these diseases to affect CKD at a population level might take another 10 years^33^.

Recently, serum cystatin C was reported to be used as an alternative to serum Cr for estimating GFR. Vega A. *et al*. compared the accuracy of the recently used equations for estimating GFR and found that cystatin C was the most accurate method^34^. In Shlipak MG’s meta-analysis, the use of cystatin C strengthened the association between the eGFR and the risks of death and end-stage renal disease^35^. However, whether GFR estimated using cystatin C is superior to that using Cr requires additional studies. Utility of cystatin C could also be added in our future researches.

The strengths of our study included the relatively large sample size (n = 22,133) and the employment of mixed model for the analyses of the association between lipid and renal function in Chinese population, and to our knowledge, this was the first study indicating the interactions between TG/HDL ratio and thyroid function on renal function. However, some limitations should also be mentioned. First, due to absence of urine sample, albuminuria was not able to be evaluated in the present study. Lack of albuminuria data might lead to misclassification of CKD, especially for those early-stage patients^32^. Therefore, it seems more appropriate to restrict our findings and conclusions to late CKD stages (3 to 5). In addition, proteinuria could lead to thyroid hormone loss with albumin, finally resulting in thyroid dysfunction. Since we investigated the modification effect of thyroid function on the relationship between TG/HDL and renal function, it was better to evaluate the urine data. Second, serum creatinine was measured using the Jaffé method and the measurements were essential to be corrected for further renal function estimation^36^. However, the Roche enzymatic assay, which is traceable to gold-standard reference methods, could not be implemented in our clinical laboratory. Therefore, the creatinine measurements were not corrected. The methods for serum creatinine measurements will be improved in our future study. Third, no distribution information of risk factors over the period of follow-up was available and thus no conclusions could be made about causal inference. Well-designed experimental research and prospective clinical studies were certainly warranted.

In conclusion, this large-scale multicenter cross-sectional study in Chinese population indicated that TG/HDL ratio was associated with renal dysfunction, and it exhibited a significantly stronger association between Cr and CKD in hypothyroidism than in euthyroidism while no significant correlation was observed in subjects with hyperthyroidism. More attention should be paid to lipid profiles to prevent or delay the occurrence and progression of renal dysfunction, especially for those with hypothyroidism. Further laboratory studies are also needed to investigate the involved complex mechanism.

## Methods

### Study participants

The present large-scale multicenter cross-sectional study was part of the REACTION study which was a national community-based program^37^,^38^,^39^,^40^. Three communities were selected from Shandong, Gansu and Jiangsu Province, including both rural and urban areas with different degrees of urbanization and economic development. All registered Han Chinese residents aged 40 years and older who had lived in their current residence for at least 5 years were invited by telephone or door-by-door visits to undergo a screening examination at the specified hospital or local health clinics. A total of 24,256 persons participated and the response rate was over 85% for each community. All participants provided an overnight fasting blood sample as well as a self-reported questionnaire. The study has been approved by the ethics committee of Shanghai Jiao Tong University^37^, and the research was carried out in accordance with this approved guidelines. Written informed consent was obtained from each participant.

We excluded subjects based on the following criteria: (1) missing vital data, such as age, gender, lipid profiles, serum Cr and thyroid function; (2) complications or conditions that affect lipid metabolism, renal function and thyroid status, such as pregnancy, lactation, malignant tumors or severe hepatic dysfunction (either or both alanine aminotransferase and aspartate aminotransferase levels above 100 U/L); and (3) taking any medicine that affects the lipid metabolism, renal or thyroid function, such as statins, fibrates, nonsteroidal anti-inflammation drugs, diuretics, thyroid hormone, anti-thyroid drugs, amiodarone or steroid hormones in the past 3 months. Finally, a total of 22,133 subjects were selected, and the excluded subjects were not significantly different from the total population in terms of age and gender.

### Data collection

All study investigators successfully completed a training program. Training sessions were standardized and scripted for all testing sites to minimize instructor variability. Weight and height were measured, with body mass index calculated as the ratio of weight/height^2^. Waist circumference was measured in centimeters. Blood pressure was the mean value of the three 3-minute interval measurements for each individual with an electronic sphygmomanometer (HEM-7117; Omron, Kyoto, Japan). Gender, age and other essential information were obtained from the standard questionnaire by trained investigators^41^.

Blood samples were collected from all of the participants between 8:00 A.M. and 10:00 A.M. after a minimum 10-hour fast. The glucose samples were measured within two hours. Serum samples were separated and shipped on dry ice. Chemiluminescent methods (Cobas E601; Roche, Basel, Switzerland) were used to quantitate thyroid function based on free tri-iodothyronine (FT_3_), free thyroxine (FT_4_) and thyrotropin (TSH) levels. The serum lipid profiles and hepatic and renal function were determined using the ARCHITECT ci16200 Integrated System (Abbott, Illinois, USA) at the central laboratory in the Shanghai Institute of Endocrine and Metabolic Diseases. The intraassay and interassay coefficients of variation were always below 5% for all above parameters.

### Definition of CKD and thyroid dysfunction

The eGFR was calculated using the simplified Modification of Diet in Renal Disease (MDRD) equation for Chinese^42^: eGFR (ml/min/1.73 m^2^) = 175 × Cr (mg/dl)^−1.234^ × age (year)^−0.179^ ×  (if, female × 0.79). CKD was defined as the eGFR below 60 ml/min/1.73 m^2^ 43.

The thyroid dysfunction was defined based on serum TSH, FT_4_ and FT_3_ levels. The laboratory reference ranges were 3.1–6.8 pmol/L for FT_3_, 12–22 pmol/L for FT_4_ and 0.27–4.2 mIU/L for TSH. Euthyroidism was defined by TSH, FT_4_ and FT_3_ levels within the reference ranges. Hyperthyroidism was a condition with decreased TSH concentration and normal or increased thyroid hormones levels including both FT_4_ and FT_3_, and hypothyroidism was defined by elevated TSH levels and normal or decreased FT_4_ levels^44^.

### Statistical analyses

All continuous variables were presented as means with standard deviations, or medians with interquartile ranges as appropriate, based on whether the distribution was normal or skewed, which was judged by histogram. Categorical data were summarized as percentage. We grouped subjects according to the development of CKD and compared continuous variables with two sample T-test or Wilcox rank sum test, and categorical variables with chi-square test.

Considering the data were obtained from different clusters (provinces), mixed model was preferred since it was better to capture the specific within-cluster correlation through adding random cluster effects into the traditional regression model. The details of the mixed model were shown in the Supplemental Materials. We conducted linear mixed model^17^ to examine the associations between TG/HDL and Cr and eGFR, while generalized linear mixed model with “logit” link function was used for CKD status^18^. In addition to TG/HDL, independent variables included in the multivariate adjusted mixed model for Cr were age, gender, body mass index, waist circumference, total cholesterol, low-density lipoprotein cholesterol, fasting plasma glucose, systolic blood pressure, diastolic blood pressure, alanine aminotransferase, aspartate aminotransferase and thyroid function status, while all factors except age and gender were adjusted for eGFR and CKD given that these two dependent variables are derived from age and gender. U-test was then adopted to compare the difference of the association between TG/HDL and renal function (regression coefficients) between euthyroidism and hypothyroidism. Furthermore, we also conducted four subgroup analyses including male, female, age less or equal to 65 and age larger than 65. Two-sided *P* values less than 0.05 were considered to be significant. All analyses were performed with R package *lme4* (http://cran.r-project.org/).

## Additional Information

**How to cite this article**: Yuan, Z. *et al*. Thyroid function modifies the association between ratio of triglyceride to high-density lipoprotein cholesterol and renal function: a multicenter cross-sectional study. *Sci. Rep*. **5**, 11052; doi: 10.1038/srep11052 (2015).

## Supplementary Material

Supplementary Information

## Figures and Tables

**Table 1:** **Clinical characteristics of subjects grouped according to the development of CKD.**

	All (n = 22,133)	CKD (n = 268)	Non-CKD (n = 21,865)	*P* value
Age (year)	56.74 ± 8.62	61.91 ± 8.33	56.67 ± 8.60	<0.001
Male (%)	35.27	19.03	35.47	<0.001
BMI (kg/m^2^)	24.70 ± 3.41	24.86 ± 3.50	24.70 ± 3.40	0.45
WC (cm)	86.37 ± 10.12	86.19 ± 10.03	86.37 ± 10.12	0.76
TC (mmol/L)	4.89 ± 1.11	5.20 ± 1.20	4.89 ± 1.10	<0.001
TG (mmol/L)	1.33 (0.99)	1.85 (1.37)	1.33 (0.99)	<0.001
LDL-C (mmol/L)	2.83 ± 0.86	3.03 ± 0.98	2.83 ± 0.86	<0.001
HDL-C (mmol/L)	1.34 ± 0.34	1.24 ± 0.28	1.34 ± 0.34	<0.001
TG/HDL	1.03 (0.97)	1.49 (1.41)	1.03 (0.97)	<0.001
FPG (mmol/L)	6.19 ± 1.84	6.94 ± 2.69	6.18 ± 1.83	<0.001
SBP (mmHg)	133.53 ± 20.58	136.13 ± 21.91	133.50 ± 20.56	0.05
DBP (mmHg)	79.00 ± 11.33	77.47 ± 11.30	79.02 ± 11.33	0.03
ALT (U/L)	18.65 ± 10.82	19.67 ± 11.23	18.64 ± 10.82	0.14
AST (U/L)	22.50 ± 8.68	23.94 ± 9.88	22.48 ± 8.67	0.02
Thyroid status (%)				
Euthyroidism	69.18	54.85	69.36	<0.001
Hyperthyroidism	3.50	2.61	3.51	0.53
Hypothyroidism	23.42	37.31	23.25	<0.001

Data are presented as means ± standard deviations, medians (interquartile ranges) or percentage. *P* values are resulted from the differences between CKD and Non-CKD participants. In addition to hyperthyroidism and hypothyroidism, other thyroid dysfunctions are not listed in the table. Abbreviations: BMI, body mass index; WC, waist circumference; TC, total cholesterol; TG, triglyceride; LDL-C, low-density lipoprotein cholesterol; HDL-C, high-density lipoprotein cholesterol; FPG, fasting plasma glucose; SBP, systolic blood pressure; DBP, diastolic blood pressure; ALT, alanine aminotransferase; AST, aspartate aminotrasferase.

**Table 2:** Association between TG/HDL and renal function using mixed model.

	β	SE	95% CI	*P*value
**Cr**				
Univariate Model	1.203	0.075	(1.056,1.350)	<0.001
Multivariate Model*	0.551	0.080	(0.394,0.708)	<0.001
**eGFR**				
Univariate Model	−1.367	0.117	(−1.596,−1.138)	<0.001
Multivariate Model*	−0.481	0.128	(−0.731,−0.230)	<0.001
**CKD**				
Univariate Model	0.131	0.026	(0.080,0.182)	<0.001
Multivariate Model*	0.173	0.034	(0.106,0.240)	<0.001

Data are coefficient (β), standard error (SE), 95% confidence interval (CI) and significance (*P* value).

*P* values are resulted from the hypothesis test whether the estimation of parameter β is significantly different from zero.

^*^Multivariate model for Cr is adjusted for age, gender, body mass index, waist circumference, total cholesterol, low-density lipoprotein cholesterol, fasting plasma glucose, systolic blood pressure, diastolic blood pressure, alanine aminotransferase, aspartate aminotrasferase and thyroid function status, while all factors except age and gender adjusted for eGFR and CKD.

**Table 3:** Interaction between TG/HDL and TSH on renal function using mixed model.

	Hyperthyroidism (n = 774)		Euthyroidism (n = 15,312)		Hypothyroidism (n = 5,183)	
	β (95%CI)	*P* value	β (95%CI)	*P* value	β (95%CI)	*P* value
**Cr**						
Univariate model	1.02 (0.27,1.76)	0.008	1.14 (0.97,1.31)	<0.001	1.46 (1.14,1.79)	<0.001
Multivariate model*	–0.20 (–0.97,0.56)	0.60	0.49 (0.31,0.67)	<0.001	**0.88**# (0.53,1.22)	<0.001
**eGFR**						
Univariate model	–0.55 (–1.63,0.52)	0.31	–1.37 (–1.64,–1.11)	<0.001	–1.48 (–1.97,–0.98)	<0.001
Multivariate model*	0.48 (–0.68, 1.64)	0.42	–0.53 (–0.82,–0.24)	<0.001	–0.51 (–1.04,0.03)	0.06
**CKD**						
Univariate model	0.11 (–0.10,0.33)	0.30	0.08 (0.001,0.16)	0.04	0.21 (0.13,0.29)	<0.001
Multivariate model*	0.08 (–0.29,0.45)	0.66	0.12 (0.02,0.23)	0.02	**0.28**# (0.17,0.40)	<0.001

Data are coefficient (β), 95% confidence interval (CI) and significance (*P* value).

*P* values are resulted from the hypothesis test whether the estimation of parameter β is significantly different from zero.

^*^Multivariable model for Cr is adjusted for age, gender, BMI, WC, TC, LDL-C, FPG, SBP, DBP, AST and ALT, while all factors except age and gender adjusted for eGFR and CKD.

^#^*P* < 0.05 compared with the coefficient of the euthyroidism group.
