# Supplementary material for: Thyroid function modifies the association between ratio of triglyceride to high-density lipoprotein cholesterol and renal function: a multicenter cross-sectional study
Source: Sci Rep. 2015 Jul 16;5:11052. doi: 10.1038/srep11052 (PMC4503948; doi:10.1038/srep11052)
Supplement: Supplementary Information [file srep11052-s1.doc]

**Thyroid function modifies the association between ratio of triglyceride to high-density lipoprotein cholesterol and renal function:**

**a multicenter cross-sectional study**

Zhongshang Yuan^1,2,3#^, Meng Zhao^2,3#^, Bingchang Zhang^4#^, Haiqing Zhang^2,3^, Xu Zhang^2,3^, Qingbo Guan^2,3^, Guang Ning^5^, Ling Gao^3,6^, Fuzhong Xue^1*^ , Jiajun Zhao^2,3*^ and the REACTION Study Group

^1^ Department of Epidemiology and Biostatistics, School of Public Health, Shandong University, Jinan, Shandong, China;

^2^ Department of Endocrinology and Metabolism, Shandong Provincial Hospital affiliated to Shandong University, Jinan, Shandong, China;

^3^ Institute of Endocrinology and Metabolic Diseases, Shandong Academy of Clinical Medicine, Jinan, Shandong, China;

^4^ Clinical Laboratory, Shandong Provincial Hospital affiliated to Shandong University, Jinan, Shandong, China;

^5^ Shanghai Clinical Center for Endocrine and Metabolic Diseases, Shanghai Institute of Endocrine and Metabolic Diseases, Department of Endocrine and Metabolic Diseases, Rui-Jin Hospital, Shanghai Jiao Tong University School of Medicine, Shanghai, China;

^6^ Scientific Center, Shandong Provincial Hospital affiliated to Shandong University, Jinan, Shandong, China.

^#^ These authors contributed equally to this study.

*Corresponding author and person to whom reprint requests should be addressed:*

Fuzhong Xue, Ph.D.

Department of Epidemiology and Biostatistics, School of Public Health, Shandong University, 44, Wenhua Xi Road, 250012, Jinan, Shandong, China.

Tel: +86-531-88380280

Fax: +86-531-88382553

E-mail: [xuefzh@sdu.edu.cn](mailto:xuefzh@sdu.edu.cn)

Jiajun Zhao, Ph.D.

Department of Endocrinology and Metabolism, Shandong Provincial Hospital affiliated to Shandong University, 324, Jingwu Road, 250021, Jinan, Shandong, China.

Tel: +86-531-68776375 (Clin.), +86-531-68776094 (Lab.)

Fax: +86-531-87068707

E-mail: [jjzhao@medmail.com.cn](mailto:jjzhao@medmail.com.cn)

**Supplemental Materials**

**Methods**

**Statistical analyses**

Considering the data were obtained from different clusters (provinces), mixed model was preferred in the manuscript. Often, the mathematic form is *y = X β + Cγ+ Z μ + ε*. *y* indicates the outcome variable, *β* is regression coefficient representing fixed effects of predictor variable *X*, *γ* is regression coefficient of potential confounding *C*, *μ* indicates random effects, *ε* indicates random errors. The interpretation of *β* is the [expected](http://en.wikipedia.org/wiki/Expected_value) change in *y* for a one-unit change in *X* given the other confounding *C* are fixed. In our manuscript, taking Cr as an example, given the other confounding factors, Cr will be expected to increase by *β* for a one-unit change in TG/HDL.

**Tables**

**Table S1. Association between TG/HDL and renal function parameters using mixed model in male**

|  | β | SE | 95% CI | *P* value |
| --- | --- | --- | --- | --- |
| **Cr** |  |  |  |  |
| Univariate Model | 0.684 | 0.108 | (0.472,0.896) | <0.001 |
| Multivariable Model^*^ | 0.408 | 0.126 | (0.162,0.654) | 0.001 |
| **eGFR** |  |  |  |  |
| Univariate Model | -0.932 | 0.177 | (-1.280,-0.584) | <0.001 |
| Multivariable Model^*^ | -0.242 | 0.204 | (-0.641,0.157) | 0.235 |
| **CKD** |  |  |  |  |
| Univariate Model | 0.075 | 0.058 | (-0.038,0.188) | 0.193 |
| Multivariable Model^*^ | 0.047 | 0.082 | (-0.114,0.208) | 0.570 |

Data are coefficient (β), standard error (SE), 95% confidence interval (CI) and significance (*P* value).

* Multivariable model for Cr is adjusted for age, BMI, WC, TC, LDL-C, FPG, SBP, DBP, AST, ALT and thyroid status, while all factors except age adjusted for eGFR and CKD.

*P* values are resulted from the hypothesis test whether the estimation of parameter β is significantly different from zero.

**Table S2. Association between TG/HDL and renal function parameters using mixed model in female**

|  | β | SE | 95% CI | *P* value |
| --- | --- | --- | --- | --- |
| **Cr** |  |  |  |  |
| Univariate Model | 1.035 | 0.092 | (0.855,1.215) | <0.001 |
| Multivariable Model^*^ | 0.583 | 0.103 | (0.381,0.785) | <0.001 |
| **eGFR** |  |  |  |  |
| Univariate Model | -1.905 | 0.152 | (-2.203,-1.606) | <0.001 |
| Multivariable Model^*^ | -0.703 | 0.161 | (-1.018,-0.387) | <0.001 |
| **CKD** |  |  |  |  |
| Univariate Model | 0.178 | 0.031 | (0.117,0.239) | <0.001 |
| Multivariable Model^*^ | 0.236 | 0.042 | (0.154,0.318) | <0.001 |

Data are coefficient (β), standard error (SE), 95% confidence interval (CI) and significance (*P* value).

* Multivariable model for Cr is adjusted for age, BMI, WC, TC, LDL-C, FPG, SBP, DBP, AST, ALT and thyroid status, while all factors except age adjusted for eGFR and CKD.

*P* values are resulted from the hypothesis test whether the estimation of parameter β is significantly different from zero.

**Table S3. Association between TG/HDL and renal function parameters using mixed model in subjects with age less or equal to 65 years**

|  | β | SE | 95% CI | *P* value |
| --- | --- | --- | --- | --- |
| **Cr** |  |  |  |  |
| Univariate Model | 1.174 | 0.080 | (1.018,1.330) | <0.001 |
| Multivariable Model^*^ | 0.406 | 0.085 | (0.238,0.574) | <0.001 |
| **eGFR** |  |  |  |  |
| Univariate Model | -1.228 | 0.125 | (-1.473,-0.982) | <0.001 |
| Multivariable Model^*^ | -0.231 | 0.138 | (-0.503,0.040) | 0.095 |
| **CKD** |  |  |  |  |
| Univariate Model | 0.130 | 0.033 | (0.066,0.195) | <0.001 |
| Multivariable Model^*^ | 0.162 | 0.042 | (0.079,0.244) | <0.001 |

Data are coefficient (β), standard error (SE), 95% confidence interval (CI) and significance (*P* value).

* Multivariable model for Cr is adjusted for age, gender, BMI, WC, TC, LDL-C, FPG, SBP, DBP, AST, ALT and thyroid status, while all factors except age and gender adjusted for eGFR and CKD.

*P* values are resulted from the hypothesis test whether the estimation of parameter β is significantly different from zero.

**Table S4. Association between TG/HDL and renal function parameters using mixed model in subjects with age older than 65 years**

|  | β | SE | 95% CI | *P* value |
| --- | --- | --- | --- | --- |
| **Cr** |  |  |  |  |
| Univariate Model | 1.475 | 0.206 | (1.071,1.879) | <0.001 |
| Multivariable Model^*^ | 1.435 | 0.218 | (1.009,1.862) | <0.001 |
| **eGFR** |  |  |  |  |
| Univariate Model | -2.423 | 0.289 | (-2.988,-1.857) | <0.001 |
| Multivariable Model^*^ | -1.703 | 0.316 | (-2.324,-1.083) | <0.001 |
| **CKD** |  |  |  |  |
| Univariate Model | 0.160 | 0.047 | (0.068,0.251) | 0.001 |
| Multivariable Model^*^ | 0.224 | 0.064 | (0.099,0.349) | <0.001 |

Data are coefficient (β), standard error (SE), 95% confidence interval (CI) and significance (*P* value).

* Multivariable model for Cr is adjusted for age, gender, BMI, WC, TC, LDL-C, FPG, SBP, DBP, AST, ALT and thyroid status, while all factors except age and gender adjusted for eGFR and CKD.

*P* values are resulted from the hypothesis test whether the estimation of parameter β is significantly different from zero.

**Table S5. Interaction between TG/HDL and TSH on renal function parameters using mixed model in male**

|  | Hyperthyroidism  (n=285) | | Euthyroidism  (n=5983) | | Hypothyroidism  (n=1155) | |
| --- | --- | --- | --- | --- | --- | --- |
|  | β(95%CI) | *P* value | β(95%CI) | *P* value | β(95%CI) | *P* value |
| **Cr** |  |  |  |  |  |  |
| Univariate model | -0.11(-1.15,0.93) | 0.83 | 0.64(0.41,0.86) | <0.001 | 0.93(0.16,1.70) | 0.02 |
| Multivariate model^*^ | -0.33(-1.58,0.93) | 0.61 | 0.35(0.09,0.61) | 0.009 | 0.63(-0.27,1.54) | 0.17 |
| **eGFR** |  |  |  |  |  |  |
| Univariate model | 0.14(-1.12,1.41) | 0.83 | -0.86(-1.26,-0.46) | <0.001 | -1.09(-2.03,-0.14) | 0.02 |
| Multivariate model^*^ | 0.77(-0.74, 2.28) | 0.32 | -0.15(-0.61,0.32) | 0.53 | -0.41(-1.51,0.70) | 0.47 |
| **CKD** |  |  |  |  |  |  |
| Univariate model | -1.09(-3.79,1.61) | 0.43 | 0.07(-0.08,0.23) | 0.34 | 0.14(-0.04,0.33) | 0.13 |
| Multivariate model^*^ | -1.41(-4.34,1.51) | 0.34 | 0.01(-0.22,0.23) | 0.96 | 0.08(-0.16,0.33) | 0.50 |

Data are coefficient (β), 95% confidence interval (CI) and significance (*P* value).

^*^ Multivariable model for Cr is adjusted for age, BMI, WC, TC, LDL-C, FPG, SBP, DBP, AST and ALT, while all factors except age adjusted for eGFR and CKD.

*P* values are resulted from the hypothesis test whether the estimation of parameter β is significantly different from zero.

**Table S6. Interaction between TG/HDL and TSH on renal function parameters using mixed model in female**

|  | Hyperthyroidism  (n=489) | | Euthyroidism  (n=9329) | | Hypothyroidism  (n=4028) | |
| --- | --- | --- | --- | --- | --- | --- |
|  | β(95%CI) | *P* value | β(95%CI) | *P* value | β(95%CI) | *P* value |
| **Cr** |  |  |  |  |  |  |
| Univariate model | 1.37(0.41,2.33) | 0.005 | 0.96(0.74,1.19) | <0.001 | 1.16(0.83,1.49) | <0.001 |
| Multivariate model^*^ | 0.34(-0.68,1.36) | 0.51 | 0.56(0.31,0.81) | <0.001 | 0.70(0.34,1.07) | 0.002 |
| **eGFR** |  |  |  |  |  |  |
| Univariate model | -2.09(-4.04,-0.15) | 0.04 | -2.05(-2.41,-1.70) | <0.001 | -1.50(-2.09,-0.91) | <0.001 |
| Multivariate model^*^ | -0.30(-2.28, 1.68) | 0.77 | -0.95(-1.33,-0.57) | <0.001 | -0.17(-0.78,0.45) | 0.60 |
| **CKD** |  |  |  |  |  |  |
| Univariate model | 0.34(0.02,0.65) | 0.04 | 0.14(0.04,0.23) | 0.004 | 0.23(0.14,0.32) | <0.001 |
| Multivariate model^*^ | 0.32(-0.35,0.99) | 0.35 | 0.22(0.10,0.33) | <0.001 | 0.31(0.18,0.45) | <0.001 |

Data are coefficient (β), 95% confidence interval (CI) and significance (*P* value).

^*^ Multivariable model for Cr is adjusted for age, BMI, WC, TC, LDL-C, FPG, SBP, DBP, AST and ALT, while all factors except age adjusted for eGFR and CKD.

*P* values are resulted from the hypothesis test whether the estimation of parameter β is significantly different from zero.

**Table S7. Interaction between TG/HDL and TSH on renal function using mixed model in subjects with age less or equal to 65 years**

|  | Hyperthyroidism  (n=582) | | Euthyroidism  (n=12754) | | Hypothyroidism  (n=4218) | |
| --- | --- | --- | --- | --- | --- | --- |
|  | β (95%CI) | *P* value | β (95%CI) | *P* value | β (95%CI) | *P* value |
| **Cr** |  |  |  |  |  |  |
| Univariate model | 1.04 (0.20,1.88) | 0.02 | 1.14 (0.96,1.32) | <0.001 | 1.26 (0.90,1.62) | <0.001 |
| Multivariate model* | -0.23 (-1.09,0.63) | 0.60 | 0.38(0.18,0.58) | <0.001 | 0.44 (0.06,0.82) | 0.02 |
| **eGFR** |  |  |  |  |  |  |
| Univariate model | -0.57 (-1.74,0.60) | 0.34 | -1.22 (-1.51,-0.93) | <0.001 | -1.18 (-1.72,-0.63) | <0.001 |
| Multivariate model* | 0.62 (-0.64, 1.88) | 0.34 | -0.29 (-0.61,0.04) | 0.08 | 0.05 (-0.54,0.64) | 0.87 |
| **CKD** |  |  |  |  |  |  |
| Univariate model | 0.09 (-0.21,0.38) | 0.56 | 0.11 (0.003,0.21) | 0.03 | 0.19 (0.09,0.29) | <0.001 |
| Multivariate model* | 0.13(-0.26,0.52) | 0.51 | 0.15 (0.03,0.27) | 0.01 | 0.24 (0.11,0.37) | <0.001 |

Data are coefficient (β), 95% confidence interval (CI) and significance (*P* value).

^*^ Multivariable model for Cr is adjusted for age, gender, BMI, WC, TC, LDL-C, FPG, SBP, DBP, AST and ALT, while all factors except age and gender adjusted for eGFR and CKD.

*P* values are resulted from the hypothesis test whether the estimation of parameter β is significantly different from zero.

**Table S8. Interaction between TG/HDL and TSH on renal function using mixed model in subjects with age older than 65 years**

|  | Hyperthyroidism  (n=774) | | Euthyroidism  (n=15,312) | | Hypothyroidism  (n=965) | |
| --- | --- | --- | --- | --- | --- | --- |
|  | β (95%CI) | *P* value | β (95%CI) | *P* value | β (95%CI) | *P* value |
| **Cr** |  |  |  |  |  |  |
| Univariate model | 0.98 (-0.64,2.59) | 0.24 | 1.19 (0.70,1.68) | <0.001 | 2.33 (1.48,3.19) | <0.001 |
| Multivariate model* | -0.40 (-2.09,1.28) | 0.64 | 1.20 (0.69,1.71) | <0.001 | **2.37^#^** (1.42,3.31) | <0.001 |
| **eGFR** |  |  |  |  |  |  |
| Univariate model | -0.80 (-3.20,1.61) | 0.54 | -2.50 (-3.18,-1.81) | <0.001 | -2.40 (-3.56,-1.23) | <0.001 |
| Multivariate model* | 0.14 (-2.56, 2.85) | 0.92 | -1.75 (-2.49,-1.01) | <0.001 | -1.79 (-3.11,-0.47) | 0.008 |
| **CKD** |  |  |  |  |  |  |
| Univariate model | 0.25 (-0.19,0.69) | 0.26 | 0.11 (-0.02,0.24) | 0.09 | 0.26 (0.10,0.43) | 0.001 |
| Multivariate model* | _ | _ | 0.20 (0.04,0.37) | 0.02 | 0.35 (0.09,0.61) | 0.008 |

Data are coefficient (β), 95% confidence interval (CI) and significance (*P* value).

^*^ Multivariable model for Cr is adjusted for age, gender, BMI, WC, TC, LDL-C, FPG, SBP, DBP, AST and ALT, while all factors except age and gender adjusted for eGFR and CKD.

*P* values are resulted from the hypothesis test whether the estimation of parameter β is significantly different from zero.

^#^ *P* < 0.05 compared with the coefficient of the euthyroidism group.
